# Monthly Record of Deaths in the City of Buffalo

**Published:** 1854-11

**Authors:** J. M. Newman

**Affiliations:** Health Physician


### ART. VI.—*Record of Mortality in the City of Buffalo, for the Month of September*, 1854. By J. M. Newman, M. D., Health Physician.

| DISEASES. | No. | Males. | Females. |
|---|---|---|---|
| Accidens, | 3 | 1 | 2 |
| Asphyxia Immersorum, | 4 | 4 | |
| Cholera Asphyxia, | 95 | 57 | 38 |
| " Infantum, | 58 | 26 | 29 |
| Colica Pictonum, | 1 | 1 | |
| Cynanche Tonsillaris, | 1 | 1 | |
| " Trachealis, | 2 | | 2 |
| " Gangrænosa, | 1 | | 1 |
| Debilitas, | 13 | 8 | 5 |
| Diarrhœa, | 28 | 11 | 15 |
| Dysenteria, | 23 | 12 | 11 |
| Eclampsia, | 16 | 8 | 7 |
| Enteritis, | 1 | | 1 |
| Febris, | 2 | 1 | 1 |
| " Biliosa, | 1 | 1 | |
| " Intermittens, | 1 | 1 | |
| " Puerperal, | 1 | | 1 |
| " Typhoid, | 7 | 4 | 3 |
| " Typhus, | 5 | 3 | 2 |
| Hæmatemesis, | 1 | 1 | |
| Hydrops, | 1 | 1 | |
| " Cerebri, | 6 | 2 | 4 |
| Hydrophobia, | 1 | 1 | |
| Hyperæmia Cerebri, | 5 | 2 | 2 |
| Icterus Malignus, | 1 | | 1 |
| Intemperantia et Delir. Tremens, | 4 | 4 | |
| Laryngitis, | 1 | | 1 |
| Marasmus, | 12 | 5 | 5 |
| Morbi Uteri, | 3 | | 3 |
| Myelitis, | 1 | | 1 |
| Natus Mortuus et Partus Abortivi, | 12 | 5 | 4 |
| Negligentia, | 1 | 1 | |
| Peritonitis, | 1 | I | |
| Phthisis Pulmonalis, | 20 | 10 | 10 |
| Phrenitis, | 3 | 3 | |
| Pertussis, | 2 | | 2 |
| Pneumonia, | 3 | 3 | |
| Purpura Hæmorrhagica, | 1 | 1 | |
| Rubeola, | 1 | | 1 |
| Scirrhus Pylori, | 1 | 1 | |
| Scrophula, | 2 | | 1 |
| Senectus, | 5 | 3 | 2 |
| Suspectus Homicidium, | 1 | 1 | |
| Tumor Cervicis, | 1 | | 1 |
| Ulceratio Intestinorum, | 1 | | 1 |
| Ignotus, | 28 | 9 | 13 |
| | 382 | | |

| | |
|---|---|
| Males, | 193 |
| Females, | 170 |
| Sex not given, | 19 |
| Total, | 382 |

REGISTER OF MORTALITY—(Continued.)

AGES.

| | | | |
|---|---|---|---|
| Still-born, | 8 | Between 6 years and 12 years, | 10 |
| 1 day, | 6 | " 12 " " 20 " | 15 |
| Between 1 day and 15 days, | 13 | " 20 " " 30 " | 51 |
| " 15 days and 30 days, | 10 | " 30 " " 40 " | 43 |
| " 1 month and 3 months, | 15 | " 40 " " 50 " | 14 |
| " 3 months and 6 months, | 19 | " 50 " " 60 " | 17 |
| " 6 " " 9 " | 21 | " 60 " " 70 " | 11 |
| " 9 " " 12 " | 30 | " 70 " " 80 " | 8 |
| " 1 year and 3 years, | 67 | " 80 " " 90 " | 1 |
| " 3 years and 6 years, | 20 | Ages not given, | 3 |
| Total, | | | 382 |

NATIVITIES.

| | | | |
|---|---|---|---|
| American, | 133 | Swiss, | 2 |
| English, | 13 | Norwegian, | 1 |
| Scotch, | 6 | Hollander, | 1 |
| Irish, | 35 | Colored, | 2 |
| Canadian, | 2 | Country not given, | 50 |
| German, | 137 | | |
| Total, | | | 382 |

Of the above number of deaths 12 were at the Almshouse.

